# Automated Single-Sperm Selection Software (SiD) during ICSI: A Prospective Sibling Oocyte Evaluation

**DOI:** 10.3390/medsci12020019

**Published:** 2024-03-27

**Authors:** Debbie Montjean, Marie-Hélène Godin Pagé, Carmen Pacios, Annabelle Calvé, Ghenima Hamiche, Moncef Benkhalifa, Pierre Miron

**Affiliations:** 1 Centre d’aide médicale à la procréation Fertilys, 1950 Maurice-Gauvin Street, Laval, QC H7S 1Z5, Canada; marie-helene.godin@fertilys.org (M.-H.G.P.); carmen.pacios@fertilys.org (C.P.);; 2 Médecine et Biologie de la Reproduction, CECOS de Picardie et Laboratoire PERITOX, Université Picardie Jules Verne, CBH-CHU Amiens Picardie, 1 Rond-Point du Professeur Christian Cabrol, 80054 Amiens, France

**Keywords:** ICSI, artificial intelligence, automated sperm selection, laboratory outcomes, pregnancy rate

## Abstract

The computer-assisted program SiD was developed to assess and select sperm in real time based on motility characteristics. To date, there are limited studies examining the correlation between AI-assisted sperm selection and ICSI outcomes. To address this limit, a total of 646 sibling MII oocytes were randomly divided into two groups as follows: the ICSI group (n = 320): ICSI performed with sperm selected by the embryologist and the ICSI-SiD group (n = 326): ICSI performed with sperm selected using SiD software. Our results show a non-significant trend towards improved outcomes in the ICSI-SiD group across various biological parameters, including fertilization, cleavage, day 3 embryo development, blastocyst development, and quality on day 5. Similarly, we observed a non-significant increase in these outcomes when comparing both groups with sperm selection performed by a junior embryologist. Embryo development was monitored using a timelapse system. Some fertilization events happen significantly earlier when SiD is used for ICSI, but no significant difference was observed in the ICSI-SiD group for other timepoints. We observed comparable cumulative early and clinical pregnancy rates after ICSI-SiD. This preliminary investigation illustrated that employing the automated sperm selection software SiD leads to comparable biological outcomes, suggesting its efficacy in sperm selection.

## 1. Introduction

Since its development in the early 1990s, intracytoplasmic sperm injection (ICSI) has been the most widely used fertilizing technique for in vitro fertilization (IVF) laboratories worldwide, and the number of ICSI cycles is constantly increasing [1,2,3]. One pivotal aspect of this procedure lies in the selection of the most optimal sperm for injection into the oocyte. Unlike natural fertilization or conventional IVF, where multiple sperm compete to fertilize the egg, ICSI involves the precise selection and direct injection of a single spermatozoon into the oocyte. This innovative method has revolutionized the landscape of assisted reproductive technology, offering hope to men with severe male factor infertility by providing them with similar prospects of achieving biological parenthood as couples facing less severe male factor infertility. Consequently, the selection of the sperm employed in this technique represents a critical step that significantly influences fertilization outcomes and subsequent embryo development [4,5,6,7].

Over the past decades, considerable efforts have been made in the development of new techniques to refine sperm selection with the hopes of improving IVF outcomes. These techniques include swim-up, density gradient centrifugation, hyaluronic binding, magnetic-activated cell sorting, surface charge Zeta potential, and high-resolution morphological sperm selection [8,9,10,11,12]. Microfluidics is one of the most recent approaches developed to improve sperm selection. These devices typically consist of microchannels and chambers designed to mimic the natural microenvironment of the female reproductive tract. Sperm are loaded into the microfluidic device, where they undergo passive or active sorting based on various physical and biochemical properties, including motility, size, shape, and surface markers [13,14]. This allows for the isolation of highly motile and morphologically normal sperm, which are more likely to successfully fertilize an egg. Given its centrifugation-free nature, this technique appears to closely mimic the natural journey of ejaculated sperm in the female genital tract. Moreover, it has been reported to select sperm with high motility and DNA integrity and yield better laboratory and clinical outcomes [12,13,14,15]. These compelling results have prompted many IVF laboratories to adopt this technique for sperm preparation. Nevertheless, there remains a lingering question of how to further push the boundaries in our methods of selecting the most optimal sperm for ICSI.

The implementation of artificial intelligence (AI) in reproductive medicine has aroused great interest in a variety of applications including single-sperm selection for ICSI [16,17,18,19,20,21]. When assessing the fertilizing capacity of spermatozoa, certain parameters are pivotal and directly correlated. These parameters include the overall morphology of the spermatozoon, comprising a normal head, midpiece, and tail. Additionally, sperm motility serves as a crucial indicator of potential, with immotile or non-progressive sperm typically excluded from consideration due to their limited fertilization potential. However, while certain characteristics may be relatively clear-cut in their assessment, others present challenges in their evaluation under the microscope. Notably, factors such as the direction and velocity of sperm movement offer valuable insights into overall function and fertilization potential [21]. However, these parameters are challenging to assess accurately through manual observation under a microscope; for instance, determining the speed of a single sperm is impractical by visual inspection alone. Consequently, the computer-assisted program SiD (Sperm ID), was designed to grade sperm, in real time, based on progressive motility parameters such as VSL (straight-line velocity), LIN (linearity of the curvilinear path), and HMP (head movement pattern). Subsequently, the program synthesizes these measurements to generate a quantitative score reflecting the quality of the parameters. SiD’s assistance is proposed to optimize the selection of the most optimal sperm for ICSI based on these three parameters. Initial investigations into this program have revealed significant differences in VSL, LIN, and HMP among SiD-selected spermatozoa. Importantly, higher SiD scores were shown to be associated with both successful fertilization and blastocyst formation [21].

The application of sperm selection by AI is expected to have numerous additional advantages. One notable advantage is its potential to mitigate user bias inherent in embryologists’ selection practices. Similarly, AI-based selection methods have the capacity to minimize interoperator variability, thereby reducing potential discrepancies in fertilization outcomes. Moreover, addressing the challenge of personnel shortages commonly encountered in laboratory settings worldwide, AI programs offer a solution to reduce the impact of user fatigue and ensure consistent selection quality, particularly in instances of limited training among personnel involved in sperm selection. Although laboratories uphold rigorous training standards, acknowledging the possibility of human variability remains imperative. Additionally, the application of AI in sperm selection has the potential to significantly optimize selection timing, further streamlining the process [21]. Despite encouraging results from this technology, there are limited studies correlating the usage of AI for sperm selection with ICSI outcomes [21]. Moreover, many still question if this technology will have a significant impact.

This prospective study aims to investigate and establish correlations between the application of AI for sperm selection and the outcomes of ICSI procedures.

## 2. Materials and Methods

### 2.1. Settings

A total of 76 patients undergoing ICSI procedures, who provided informed and signed consent, were included in this study. The patients had a mean female age of 34.9 years (±5.6) and a mean male age of 36.7 years (±6.6). Fresh and frozen sperm samples were included in this study. Fresh sperm samples were collected by masturbation, and the sample was liquified for 30 min followed by semen analysis. As for frozen sperm samples, the frozen vial of sperm was incubated at 37 °C for 5 min to allow liquefaction. Both fresh and post-thaw frozen samples underwent microscopic evaluation, assessing sperm concentration, total sperm count, and motility using a Makler counting chamber. The mean native concentration of semen samples in this study was 63.8 ± 49.2 million/mL, and the mean total motility was 58.6 ± 18.3%. Fresh and frozen sperm samples were prepared using a microfluidic chamber (Zymot™) following the manufacturer’s recommendations. Briefly, 850 μL of the fresh semen sample or 850 µL of a 1:1 dilution of the post-thaw frozen sample was inserted into the inlet port of the Zymot^TM^ system. Once the sample was inserted into the system, 750 uL of Spermwash® was placed on top of the system’s membrane. The loaded system was then enclosed in a Petri dish, and placed at 37 °C for 30 min, or for the time required to obtain at least 1 million mobile sperm/mL. After incubation, 100 μL of the sample was taken from the outlet port of the system and the sample concentration and motility were once again assessed using a Makler counting chamber. The mean post-preparation sperm concentration was 21.4 ± 24.2 million/mL and the mean total motility was 91.2 ± 8.1%. ICSI was performed following standard procedures using an inverted microscope (Zeiss Axio Observer A.1, Camera Watec 221S). Spermatozoa were released in a PVP droplet to reduce global sperm motility. Over a 1-year period, 646 sibling MII oocytes were randomly divided into the following two groups: 1—ICSI group (n = 320): ICSI performed with sperm selected by the embryologist, and 2—ICSI-SiD group (n = 326): ICSI performed with sperm selected using SiD software. Sperm morphology screening was conducted prior to the ICSI procedure in both study groups. Additionally, the level of experience of the embryologists was factored into the sperm selection process. An embryologist with less than five years of experience in clinical embryology was classified as a junior, while those with more than five years of experience were considered senior embryologists. Embryos were cultured for up to 6 days in the same conditions in ESCO MIRI^®^ incubators (SAGE 1-step™ Medium, 37.0 °C, 5%O_2_, 6% CO_2_). Our laboratory is equipped with one timelapse incubator (ESCOMIRI^®^TL). As room is limited in the timelapse incubator, only a subset of the patients had their embryos cultured and monitored within the timelapse system. There was no selection nor indication of time-lapse culture. Only single embryo transfers were performed on day 5. Surplus embryos were vitrified for future use.

### 2.2. Automated Sperm Selection Software

**Figure 1:**
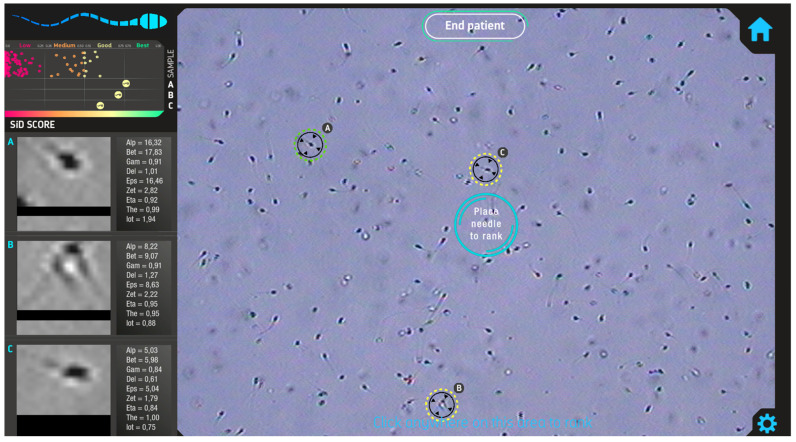
SiD software user interface. Analyzed sperms are encircled by the system and scored as «Low», «Medium», «Good», or «Best» on a red-to-green scale (upper left). The software suggests that priority for injection should be given to sperm A, then B and C.

Each sperm sample underwent video recording using an inverted microscope equipped with a digital camera (Watec 221S, Watec®, Decatur, GA, USA). Videos captured the movement of spermatozoa from the moment of introduction into the PVP droplet until the manual selection and immobilization of a spermatozoon for injection. Quantitative analysis of sperm motility parameters was performed using SiD (RV1.0.0, IVF 2.0 LTD) software. SiD computed individual values for parameters such as LIN and VSL for every spermatozoon observed in the field of view and generated a quantitative score and ranking as «Low», «Medium», «Good», and «Best» (Figure 1). Sperm categorized as “Best” by the SID software were prioritized for ICSI.

### 2.3. Embryo Vitrification and Warming

Embryos were individually vitrified on a Cryotop^®^ device (Kitazato®, Fuji, Japan) using a Kitazato vitrification kit following the supplier’s recommendation. Embryos were dehydrated in equilibration solution for 10 min, exposed to vitrification solution for 1 min, placed on the Cryotop^®,^ and then directly immersed into liquid nitrogen within 1 min. Embryo-warming cycles were performed using the Kitazato warming kit. The thawing solution was warmed up at 37 °C. The strip of the Cryotop^®^ was immersed into it, and the detached embryos were incubated for 1 min. At room temperature, the embryos were transferred into the diluent solution and washed twice in droplets of wash solution. The embryos were incubated in SAGE 1-step™ Medium (37.0 °C, 5% O_2_, 6% CO_2_) at least 1 h before transfer, and embryo re-expansion, morphology, and viability were assessed before transfer. The vitrified/warmed embryos were transferred in substitute hormonal treatment for endometrial preparation.

### 2.4. Outcome Definitions

Laboratory outcomes were calculated as described in the Vienna consensus [22]. Embryos displaying less than 10% fragmentation with 4 or 8 cells were considered top-quality embryos on day 2 or day 3, respectively. Blastocyst-stage embryos were graded using the Gardner system [23]. Top-quality blastocysts showed grade A in both trophectoderm and inner cell mass and good-quality blastocysts showed grade A or B trophectoderm and inner cell mass. Embryo development was monitored using a timelapse system (MIRI^®^, ESCO, Egaa, Denmark), and embryo morphokinetic parameters were annotated manually following the nomenclature previously described [24,25]. The morphokinetics events were annotated only when they could be clearly visualized by the operator. To minimize observer bias in embryo grading and morphokinetic annotation, the method of sperm selection was blinded. Preimplantation genetic testing for aneuploidy (PGT-A) was performed to examine chromosomal abnormalities. Our clinic policy recommends PGTA testing only for the following 3 indications: 1—history of recurrent implantation failure (failure to achieve a pregnancy after 3 transfers of good quality blastocysts); 2—history of repeated pregnancy loss (3 early or 2 clinical miscarriages); and 3—women with advanced maternal age (≥37 years of age). Therefore, only a subset of our patients benefitted from this test. Early pregnancies were assessed by blood hCG detection (>10 U/L). Clinical pregnancies were confirmed by ultrasound assessment of a fetal heartbeat.

### 2.5. Statistics

Qualitative variables are expressed as percentages and quantitative variables as means with standard deviations. Student’s t-test was used to compare quantitative variables and Pearson chi-square or Fisher’s exact test was used for qualitative variables.

## 3. Results

### 3.1. Laboratory Outcomes

There was a non-significant trend towards better outcomes in the ICSI-SiD group for biological outcomes including fertilization rate, cleavage rate, day 3 embryo development rate, blastocyst development rate on day 5, good-quality blastocyst development rate on day 5, and top-quality blastocyst development rate on day 5 (Table 1).

**Figure 2:**
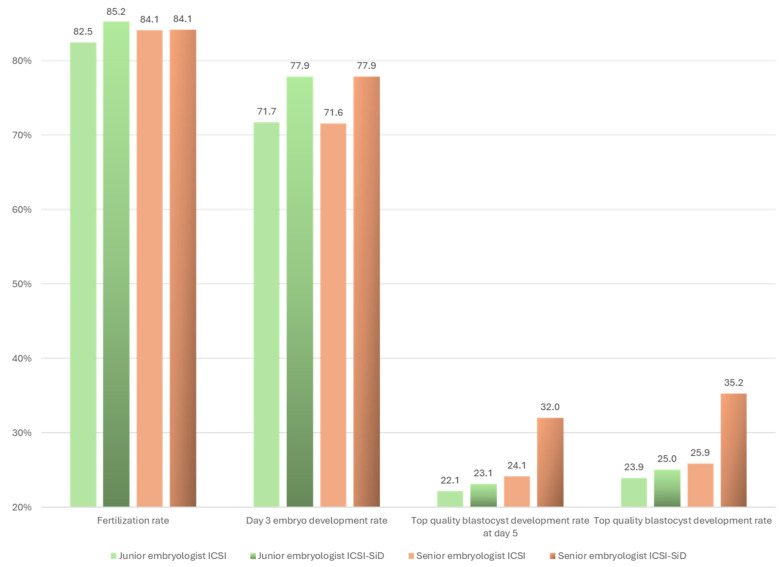
ICSI and ICSI-SiD outcomes based on the experience of the embryologist. An embryologist with less that 5 years of practice in clinical embryology was considered as a junior. Junior embryologists injected a total of 285 oocytes (ICSI-SiD = 135 and ICSI = 150) and senior embryologists performed ICSI in a total of 361 oocytes (ICSI-SiD = 191 and ICSI = 170) No statistically significant difference was observed.

Similarly, a non-significant increase was observed in all biological outcomes including fertilization rate, cleavage rate, day 3 embryo development rate, blastocyst development rate on day 5, good-quality blastocyst development rate on day 5, and top-quality blastocyst development rate on day 5 when sperm selection was performed by a junior embryologist (Figure 2).

### 3.2. Embryo Morphokinetics

**Figure 3:**
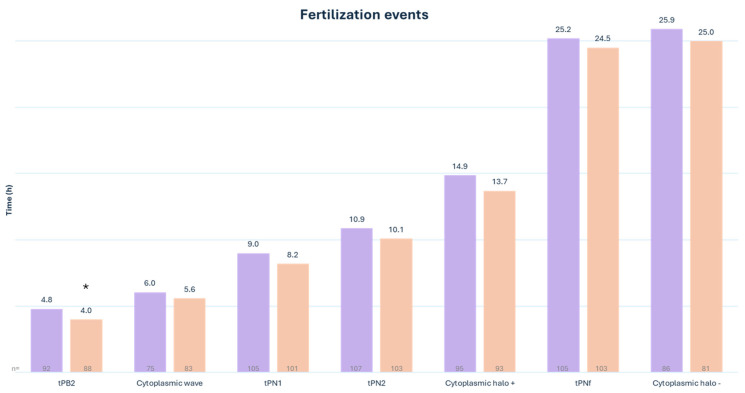
Timing of fertilization events in the ICSI (purple) group compared to the ICSI-SiD (orange) group. tPB2: time to extrusion of the 2nd polar body. tPN1-2: time to PN appearance. tPNf: time to PN fading * *p* = 0.005. n = number of visualized and annotated events.

**Figure 4:**
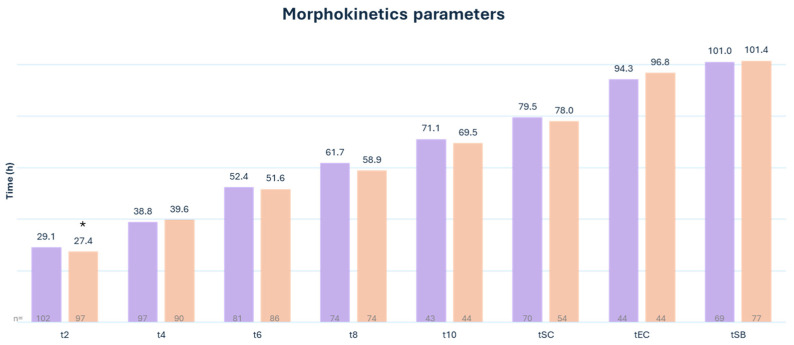
Morphokinetic parameters in the ICSI group (purple) as compared to the ICSI-SiD (orange) group. tn: time to *n* number of blastomeres. tSC: time to start of compaction. tEC: time to end of compaction. tSD: time to start of blastulation * *p* = 0.05. n = number of visualized and annotated events.

Embryo development was monitored using a timelapse system. Our data showed that second polar body extrusion (tPB2) (4.0 h vs. 4.8 h [*p* = 0.005]) (Figure 3) and early cleavage (t2) (27.3 h vs. 28.7 h [*p* = 0.05]) (Figure 4) happen significantly earlier when SiD is used for ICSI. No significant difference was observed in all other fertilization events (cytoplasmic wave, tPN1, tPN2, presence of cytoplasmic halo, tPNf, and cytoplasmic halo disappearance), the other cleavage timings (t3-> t10, tM, tSC, tEC, and tSB), or embryonic cell cycles (ECC1, ECC2, ECC3, s2, and s3).

### 3.3. Genetic and Clinical Outcomes

The euploid rate obtained within the two groups was comparable. Although there was a tendency toward higher cumulative early and clinical pregnancy rates after ICSI-SiD, the difference did not reach significance (Table 2).

## 4. Discussion

This study sought to explore the potential benefits of incorporating AI for optimizing sperm selection in the context of ICSI. In this preliminary investigation involving sibling oocytes, the comparison between automated sperm selection and embryologist-performed selection for ICSI revealed similar laboratory outcomes. Key parameters such as the fertilization rate, cleavage rate, day 3 embryo development rate, blastocyst development rate on day 5, good-quality blastocyst development rate on day 5, and top-quality blastocyst development rate on day 5 exhibited a non-significant trend towards improved outcomes with the use of the automated software. Similarly, clinical outcomes, including the cumulative early pregnancy rate and cumulative clinical pregnancy rate, demonstrated a positive non-significant trend when the software was involved in sperm selection.

One potential contributing factor to the observed lack of difference could be attributed to the methodology employed for sperm sample preparation. In this study, the Zymot^TM^ microfluidic chamber was utilized for sperm preparation, which is a device recognized for optimizing sperm selection and generating a pre-optimized population of spermatozoa [3,12,14,15]. Exploring the impact of SiD automated sperm selection software on biological outcomes in samples prepared using less effective techniques may reveal divergent results in future investigations. Despite many outcomes demonstrating a positive trend without reaching statistical significance, conclusive insights into the contribution of SiD to both laboratory and clinical outcomes may necessitate larger datasets for a definitive conclusion.

Upon further analysis based on embryologist experience, slightly higher non-significant biological outcomes were noted when the AI software selected sperm for both junior and senior embryologists. Importantly, no significant difference emerged in the outcomes when comparing sperm selection by junior and senior embryologists, indicating that junior embryologists in the laboratory were highly trained from the outset. These results suggest that the AI program performed on par with an embryologist in choosing the most optimal sperm for ICSI. This congruence in outcomes is particularly promising, as it indicates the AI program’s efficacy. This is especially noteworthy considering the inherent complexity of evaluating a sperm’s potential with the naked eye of an embryologist. The embryologist must assess various aspects of sperm mobility, as well as multiple facets of sperm morphology, encompassing the head, midpiece, and tail. All these evaluations must be conducted while numerous sperm are present in the same field of vision, each exhibiting distinct movements. The use of AI in sperm selection addresses these challenges, providing a standardized and objective approach that overcomes the intricacies associated with manual assessments. Moreover, the AI tool’s ability to eliminate user bias, mitigate fatigue, and optimize time management adds significant value to the ICSI process, especially in scenarios where staff may not be well-trained in sperm selection. Although our study did not directly measure and compare the time taken by an embryologist to select the most optimal sperm versus the AI program, the program’s efficiency was notably observed. Interestingly, future versions of SiD will include automated sperm morphology assessments. This improvement is expected to have a positive impact on the time taken to select sperm for ICSI. A similar study should be conducted to re-assess updated versions of the software. It must be kept in mind that evaluating the cost-effectiveness of AI-driven sperm selection is imperative for assessing its viability in clinical settings. Conducting an analysis of potential economic benefits, such as reduced laboratory staffing expenses, shorter procedure durations, and enhanced resource allocation, can provide valuable insights into its long-term viability and sustainability within the field of reproductive medicine.

In our investigation into the impact of sperm selection on embryo morphokinetics, our findings revealed that the extrusion of the second polar body (tPB2) and the first cleavage to form a two-cell embryo (t2) occurred earlier when SiD was employed for sperm selection. These specific timing events, i.e., tPB2 and t2, have been associated with critical aspects such as embryo implantation, day 3 embryo quality, and blastocyst formation and quality, as documented in previous studies [24,26,27,28,29,30]. Previous research has shown that embryos falling within the time range of 21.3 h < t2 < 27.9 h exhibit higher implantation rates compared with those outside this period [29,30]. Not all incubators available in our laboratory are equipped with a timelapse system to monitor embryo development. This explains the gap between the number of oocytes in each group (ICSI-SiD and ICSI) and the number of visualized and annotated morphokinetic events. To draw more definitive conclusions about the impact of automated sperm selection using SiD on the tPB2 and t2 timings, further investigation with a larger dataset is needed. We believe that an in-depth analysis of sperm selected by SiD may help us to interpret this finding.

We do not recommend PGT testing for routine clinical use. Therefore, we performed such analysis only in a subset of our patients with advanced maternal age or with a history of recurrent implantation failure or a history of repeated miscarriages. Consequently, only a limited number of embryos were analyzed in each group and included in the present study (15 embryos in the ICSI-SiD group and 9 embryos in the ICSI group). Although our preliminary results are informative, no definitive conclusion can be drawn yet about the input of SiD in the chromosomal status of developing embryos. The group numbers need to be increased to consider the results as robust.

It is widely recognized that IVF outcomes are influenced by patient-related factors (e.g., patient age, oocyte quality, etc.) that need to be controlled while performing statistical analyses [31,32,33]. We designed this study with sibling oocytes to mitigate the impact of such confounding factors on our results. However, we acknowledge although that endometrial preparation prior to embryo transfer was standardized, endometrial receptivity and thickness were not considered in our analyses. Clinical outcomes should be interpreted carefully taking into consideration intraindividual intercycle variability in endometrial receptivity.

## 5. Conclusions

This pilot study underscores the effectiveness of the automated sperm selection software SiD by demonstrating comparable biological outcomes and embryo morphokinetics to traditional methods. The findings suggest that SiD could be a valuable tool for standardizing sperm selection processes, particularly in environments with varying levels of laboratory staff experience. The consistent results in embryo development timelines and key biological parameters highlight SiD’s reliability in selecting sperm for successful fertilization and embryo development. While acknowledging the preliminary nature of this study, these promising results encourage further exploration of SiD’s potential applications in larger datasets. This automated tool has the potential to streamline and optimize sperm selection in assisted reproductive technologies, contributing to overall procedure quality and efficiency. Automated sperm selection is an emerging technology that appears to be a great asset in the development of the IVF laboratory of the future.

## Figures and Tables

**Table 1:** Laboratory outcomes in the ICSI-SiD group (n = 326) compared to the ICSI group (n = 320). * Includes day 5 and day 6 embryos, ns: non-significant. OR: odds ratio, CI; confidence interval.

Outcome (%)	ICSI-SiD	ICSI	OR	95% CI	*p*-Value
Fertilization rate	83.1	82.4	1.1	0.7–1.6	ns
Cleavage rate	97.6	97.2	1.2	0.4–3.7	ns
Day 2 embryo development rate	70.6	74.6	0.8	0.5–1.2	ns
Top-quality development rate on day 2	48.6	52.8	0.9	0.6–1.2	ns
Day 3 embryo development rate	72.9	70.6	1.1	0.8–1.7	ns
Top-quality embryo development rate on day 3	51.4	51.6	1.0	0.7–1.4	ns
Blastocyst development rate on day 5	49.0	44.8	1.2	0.8–1.7	ns
Good-quality blastocyst development rate on day 5	45.1	41.5	1.2	0.8–1.7	ns
Top-quality blastocyst development rate on day 5	25.9	22.2	1.2	0.8–1.9	ns
Blastocyst development rate *	70.2	62.5	1.4	1.0–2.0	ns
Good-quality blastocyst development rate *	57.3	53.6	1.1	0.8–1.7	ns
Top-quality blastocyst development rate *	29.0	24.2	1.3	0.9–1.9	ns

**Table 2:** Chromosomal and clinical outcomes in the ICSI-SiD group compared to the ICSI group. ns: non-significant. OR: odds ratio, CI: confidence interval. * Cumulates pregnancies from fresh and frozen embryo transfers. ^#^ A total number of 15 embryos were biopsied in the ICSI-SiD group and 9 embryos in the ICSI group.

Outcome (%)	ICSI-SiD	ICSI	OR	95% CI	*p*-Value
Number of embryos transferred	51	47			
Euploid rate ^#^	53.3	44.4	2.3	0.4–12.8	ns
Cumulative * early pregnancy rate	43.1	34.0	1.5	0.7–3.3	ns
Cumulative * clinical pregnancy rate	27.4	21.3	1.4	0.6–3.6	ns

## Data Availability

The data presented in this study are available on request from the corresponding author.
